# AGING-RELATED IMMUNE CELLS PHENOTYPES AND ALL-CAUSE MORTALITY IN THE FRAMINGHAM HEART STUDY

**DOI:** 10.1093/geroni/igad104.2776

**Published:** 2023-12-21

**Authors:** Ahmed A Y Ragab, Margaret Doyle, Jiachen Chen, Yuan Fang, Kathryn Lunetta, Joanne Murabito

**Affiliations:** Boston University School of Public Health, Boston, Massachusetts, United States; University of Vermont, Burlington, Vermont, United States; Boston University School of Public Health, Boston, Massachusetts, United States; Binghamton University, Binghamton, New York, United States; Boston University School of Public Health, Boston, Massachusetts, United States; Boston University Chobanian & Avedisian School of Medicine, Boston, Massachusetts, United States

## Abstract

Almost 2 billion people will likely be above the age of 60 by 2050. Aging is a process that is intrinsically complicated. One of the measures for immune system senescence is aging related immune cells phenotypes (ARIP)s. Besides CD8/CD4 ratio, new immunosenescence phenotypes have been proposed. The associations between ARIPs and all-cause mortality during long-term follow up is understudied. We profiled immune cells using flow cytometry and prospectively investigated ten different ARIPs, namely, CD4+CD27-, CD4+CD28-CD27-, CD8+CD27-, CD8+CD28-CD27-, CD4+/CD8+ ratio, CD4+(Tnaive/(T Central Memory (Tcm)+T Effector Memory (Tem)+T Effector (Teff)), CD8+ (Tnaive/(Tcm+Tem+Teff)), Granzyme B+CD8/Granzyme B+CD4 ratio, CD8+ Tc17/Treg ratio and CD4+ Th17/Treg ratio in relation to survival outcome among 990 dementia-free Framingham Heart Study (FHS) Offspring cohort participants who attended the seventh exam (1998-2001, mean age 62 years, range 40-88, 52% female). Cox proportional hazards regression models adjusting for age and sex with robust variance to account for family correlation were used to test for association between the ARIPs and hazard of death. During up to 20 years of follow up, the survival rate was 66%. Higher CD8+ Tc17/Treg ratio was significantly associated with better survival (HR:0.82 [0.7-0.94], p< 0.001). Higher CD4+ Th17/Treg ratio and CD4/CD8 ratio were also nominally associated with lower risk of death (HR:0.87, [0.75-0.97], p=0.01 and HR 0.9, [0.79-0.99], p=0.04, respectively). Other ARIPs were unassociated with all-cause mortality. We conclude that further investigation of the CD8+Tc17/Treg and CD4+ Th17/Treg ratios as ARIP biomarkers for risk of all-cause mortality is justified.

